# Benefit of primary percutaneous coronary interventions in the elderly with ST segment elevation myocardial infarction

**DOI:** 10.1136/openhrt-2019-001169

**Published:** 2020-08-03

**Authors:** Daniel Fernández-Bergés, Irene R Degano, Reyes Gonzalez Fernandez, Isaac Subirana, Joan Vila, Manuel Jiménez-Navarro, Silvia Perez-Fernandez, Mercé Roqué, Antoni Bayes-Genis, Francisco Fernandez-Aviles, Antonio Mayorga, Vicente Bertomeu-Gonzalez, Juan Sanchis, Marcos Rodríguez Esteban, Antonio Sanchez-Hidalgo, Esther Sanchez-Insa, Ane Elorriaga, Emad Abu Assi, Alberto Nuñez, Jose Manuel Garcia Ruiz, Pedro Morrondo Valdeolmillos, Daniel Bosch-Portell, Iñaki Lekuona, Andres Carrillo-Lopez, Alberto Zamora, Berta Vega-Hernandez, Javier Alameda Serrano, Catalina Rubert, Luis Ruiz-Valdepeñas, Laura Quintas, Luis Rodríguez-Padial, Jessica Vaquero, Luis Martinez Dolz, Jose A Barrabes, Pedro L Sanchez, Alessandro Sionis, Julio Martí-Almor, Roberto Elosua, Rosa-María Lidon, David Garcia-Dorado, Jaume Marrugat, on behalf of the ATHOS investigators

**Affiliations:** 1 Unidad de Investigación, Hospital Don Benito-Villanueva, Don Benito, Spain; 2 Instituto Universitario de Investigación Biosanitaria de Extremadura, Badajoz, Spain; 3 Institut Hospital del Mar d' Investigacions Mediques, Barcelona, Spain; 4 Department of Cardiology, Infanta Cristina University Hospital, Badajoz, Spain; 5 CIBERESP de Epidemiologia y Salud Publica, Barcelona, Spain; 6 Cardiology Department, Virgen de la Victoria University Hospital, Málaga, Spain; 7 CIBER Enfermedades Cardiovasculares (CIBERCV), Barcelona, Spain; 8 Institut Hospital del Mar d'Investigacions Mediques, Barcelona, Spain; 9 Hospital Clinic, Barcelona, Spain; 10 Department of Cardiology, Hospital Universitari Germans Trias i Pujol, Badalona, Spain; 11 Servicio de Cardiologia, University Hospital Gregorio Marañon, Madrid, Spain; 12 Department of Cardiology, Hospital Universitario Gregorio Marañón, Madrid, Madrid, Spain; 13 Department of Cardiology, Hospital Universitario de San Juan, Alicante, Spain; 14 Cardiology Department, Hospital Clinico Universitario, INCLIVA, Universitat de València, Valencia, Spain; 15 Department of Cardiology, Hospital Universitario Nuestra Senora de la Candelaria, Santa Cruz de Tenerife, Spain; 16 Consorci Sanitari de Terrassa, Terrassa, Spain; 17 Department of Cardiology, Hospital Universitario Miguel Servet, Zaragoza, Spain; 18 Department of Cardiology, Hospital Universitario de Basurto, Bilbao, Bilbao, Spain; 19 Department of Cardiology, Hospital Álvaro Junqueiro de Vigo, Pontevedra, Vigo, Spain; 20 Department of Cardiology, Hospital Universitario Fundación de Alcorcón, Madrid, Spain; 21 Hospital Universitario Central de Asturias, Oviedo, Spain; 22 Medicina Intensiva, Hospital Universitario Donostia, San Sebastián, San Sebastian, Spain; 23 Department of Cardiology, Hospital Universitari de Girona Doctor Josep Trueta, Girona, Spain; 24 Department of Cardiology, Hospital Galdakao-Usansolo, Galdacano, Spain; 25 Hospital Universitari Son Espases, Palma de Mallorca, Spain; 26 Hospital Comarcal de Blanes, Blanes, Spain; 27 Hospital Universitario de Cabueñes, Gijon, Spain; 28 Cardiología, Hospital Clínico Universitario Lozano Blesa, Zaragoza, Spain; 29 Hospital Son Llàtzer, Palma de Mallorca, Spain; 30 Hospital General Universitario de Ciudad Real, Ciudad Real, Spain; 31 Hospital de Mendaro, Mendaro, Spain; 32 Complejo Hospitalario de Toledo, Toledo, Spain; 33 Hospital Universitario Araba sede Txagorritxu, Vitoria-Gasteiz, Spain; 34 Hospital Universitario La Fe, Valencia, Spain; 35 Department of Cardiology, University Hospital Vall d'Hebron, Barcelona, Spain; 36 Department of Cardiology, Hospital Universitario Salamanca, Salamanca, Spain; 37 Intensive Cardiac Care Unit, Cardiology Department, Hospital de la Santa Creu i Sant Pau, Biomedical Research Institute IIB-Sant Pau, Universitat de Barcelona, Barcelona, Spain; 38 Department of Medicine, Department of Cardiology, Hospital del Mar. Universitat Autónoma de Barcelona, Barcelona, Spain; 39 Universitat de Vic, Barcelona, Spain; 40 Hospital Universitari Vall d' Hebron, Barcelona, Spain

**Keywords:** coronary intervention (PCI), stemi, acute coronary syndrome

## Abstract

**Objective:**

Primary percutaneous coronary intervention (P-PCI) has demonstrated its efficacy in patients with ST segment elevation myocardial infarction (STEMI). However, patients with STEMI ≥75 years receive less P-PCI than younger patients despite their higher in-hospital morbimortality. The objective of this analysis was to determine the effectiveness of P-PCI in patients with STEMI ≥75 years.

**Methods:**

We included 979 patients with STEMI ≥75 years, from the *ATención HOspitalaria del Síndrome coronario* study, a registry of 8142 consecutive patients with acute coronary syndrome admitted at 31 Spanish hospitals in 2014–2016. We calculated a propensity score (PS) for the indication of P-PCI. Patients that received or not P-PCI were matched by PS. Using logistic regression, we compared the effectiveness of performing P-PCI versus non-performance for the composite primary event, which included death, reinfarction, acute pulmonary oedema or cardiogenic shock during hospitalisation.

**Results:**

Of the included patients, 81.5 % received P-PCI. The matching provided two groups of 169 patients with and without P-PCI. Compared with its non-performance, P-PCI presented a composite event OR adjusted by PS of 0.55 (95% CI 0.34 to 0.89).

**Conclusions:**

Receiving a P-PCI was significantly associated with a reduced risk of major intrahospital complications in patients with STEMI aged 75 years or older.

**Key questions**

**What is already known about this subject?**

- Primary percutaneous coronary intervention (P-PCI) significantly decreases mortality of patients presenting with ST elevation myocardial infarction (STEMI) and constitutes a type I A indication in the international clinical guidelines for the treatment of acute coronary syndrome (ACS).
- Despite this recommendation, patients aged ≥75 years receives less PCI-P to the high prevalence of comorbidities (eg, like chronic renal failure or chronic obstructive pulmonary disease) and increased risk of bleeding and other complications.

**What does this study add?**

- Our study points out the significant decrease of adverse events during the hospitalisation of patients aged ≥75 years with STEMI when undergoing P-PCI in spite of their comorbidities.

**How might this impact on clinical practice?**

- Our results should extend the indication of PCI to patients aged ≥75 years to reduce their mortality when presenting a STEMI.

## Introduction

Coronary heart disease is the leading individual cause of death in Spain as in all Europe. Although mortality from coronary heart disease is decreasing, the number of events is increasing due to the ageing of the population,^1^ with an expected increase in acute coronary syndrome (ACS) in the population aged 75 years or older.^2^ The National Institute of Statistics estimates the Spanish population of ≥75 years in 2012 in more than 4 million (9%) and expects that this figure will increase—if the estimated demographic growth is maintained—up to 23% of the total population in 2052.^3^ At present, different series place the prevalence of ACS in this population above 30%, exceeding 40% in some national series.^4^

The treatment of ST segment elevation myocardial infarction (STEMI) includes performing a percutaneous coronary intervention (PCI) as soon as possible called primary PCI (P-PCI) with indication I-A (I = evidence and /or general agreement that a given treatment or procedure is beneficial, useful, effective. A = data derived from multiple randomized clinical trial or large non-randomized studies).^5^ If P-PCI is not available within 120 min from the first medical contact, fibrinolysis should be performed, except for contraindications, with subsequent angiographic study and, if possible, elective PCI (I-A). According to the most recent guide, there is no upper age limit for the recommendation of PCI, and therefore, all patients who meet the criteria regardless of their age should receive it.^5^ However, the utility of P-PCI in patients with STEMI ≥75 years is discussed, and this population receives fewer reperfusion treatments, both invasive and pharmacological.^6^ The lower reperfusion of elderly patients is probably due to the presence of atypical symptoms and to the delay in diagnosis, to their frequent comorbidity and to the higher rate of complications they may present (contrast nephropathy, major bleedings and so on).^5–7^

The lower invasive treatment in patients with STEMI ≥75 years is also due to the fact that randomised clinical trials usually do not include older patients, and if they do, they are highly selected. Therefore, the claim of the 2007 American Heart Association 7 referring to the need for clinical studies that include patients over 65 years has not lost its validity.

Our objective in the present study was to analyse whether the performance of a P-PCI reduces complications (death, reinfarction, acute pulmonary oedema or cardiogenic shock) during hospitalisation in patients with STEMI ≥75 years compared with patients with the same characteristics to which P-PCI was not performed.

## Methods

### Design

A prospective, consecutive, multicentric and national ACS registry was designed: the *ATención HOspitalaria del Síndrome coronario* (*ATHOS*) registry.^8^ This registry included 8142 patients diagnosed with ACS admitted consecutively in 31 Spanish hospitals during 2014–2016. For the present study, patients from the ATHOS registry who had an STEMI on admission and who were ≥75 years old (n=979 patients) were selected.

### Patient and public involvement

Patients or the public were not involved in the design, or conduct, or reporting, or dissemination of our research

### Study variables

Age, sex, tobacco consumption, history of diabetes, hypertension, cardiovascular disease (myocardial infarction, angina, heart failure, stroke, PCI and coronary artery bypass surgery) and related comorbidities (chronic obstructive pulmonary disease and chronic renal failure (CRF)) were recorded. Glycaemia and creatinine were collected in the admission analytical test. From the creatinine value, the estimated glomerular filtration rate (GFR) was calculated in mL/min/1.73 m^2^ using the formula Modification of Diet in Renal disease.

During hospitalisation, coronary angiography and P-PCI were recorded, as well as the prescription of medication (antiplatelet agents, heparin, ACE inhibitors, beta-blockers and diuretics). The onset of heart failure (acute pulmonary oedema and cardiogenic shock) was recorded at admission and during hospitalisation by the Killip and Kimball classification.^9^ As major bleeding complications, the fall of >3 g of haemoglobin, the need for transfusion or surgery and intracranial bleeding were considered.

### Events of interest

The primary event consisted of a composite variable that included death, reinfarction, acute pulmonary oedema or cardiogenic shock during hospitalisation.

### Statistical analysis

For the description of the continuous variables, the mean and SD were used when the variable followed a normal distribution and the median and IQR were used otherwise. Categorical variables were described as percentages. Comparisons of continuous variables between groups according to the performance of P-PCI were performed using the Student’s t-test or the Mann-Whitney test if the distribution was not normal. Categorical variables were compared between the same groups using the χ^2^ test.

To evaluate the effect of P-PCI on the primary endpoint (death, reinfarction or Killip III-IV during hospitalisation), a logistic regression model was used in patients matched by propensity score (PS) to receive P-PCI. A PS was calculated for P-PCI using a logistic regression model. For the PS model, all variables associated with performing P-PCI were included as independent variables and P-PCI as a dependent variable. Subsequently, patients with and without P-PCI were paired from the logit-PS with a tolerance of 0.2 times their SD.^10^ After the pairing of the patients, the standardised differences of the variables between paired patients with and without P-PCI were analysed. It was assumed that the variables were balanced if the standardised differences were <10.^11^ Finally, we included the paired patients in a logistic regression model, in which the dependent variable was the event of interest, and the independent variables were the P-PCI performance and the variables that were not well balanced in the process of matching by PS.

All analyses were performed with the statistical programme R (V.3.2.3).

## Results

Table 1 showed the characteristics and evolution in admission of the 979 patients aged ≥75 years with STEMI included in the analysis. P-PCI was performed in 798 (81.5%) of these patients. We observed that compared with patients who did not receive a P-PCI, the patients who did receive it had significantly younger age, higher prevalence of tobacco consumption and lower prevalence of angina, heart failure, stroke and previous CRF. On admission, patients who received P-PCI had a lower degree of heart failure and a lower prevalence of glomerular filtration rate <60 mL/min/1.73 m^2^.

**Table 1:** Characteristics of patients aged 75 years and older

	Total n=979	No P-PCI n=181	P-PCI n=798	P value
Age	83.1 (6.01)	85.0 (6.20)	82.7 (5.89)	<0.001
Sex women (%)	39.8	46.4	38.3	0.055
Killip III-IV admission (%)	15.3	27.6	12.5	<0.001
GFR <60 mL/min (%)	42.4	52.3	40.1	0.004
Glucose <126 mg/dL (%)	65.4	68.2	64.7	0.432
Diabetes mellitus (%)				0.668
No	65.7	68.0	65.2	
Yes, diet control	3.47	2.76	3.63	
Yes, OADs control	21.5	18.8	22.1	
Yes, insulin control	8.89	10.5	8.52	
Yes, diabetes in hospitalisation	0.51	0.00	0.63	
Smoker (%)				<0.004
Never	64.5	75.1	62.0	
Actual	11.8	8.29	12.7	
Ex-smoker >30 days	23.7	16.6	25.3	
Arterial hypertension	73.0	75.1	72.6	0.539
Previous MI	14.6	14.4	14.7	1.000
Previous angina	11.6	16.6	10.5	0.031
Previous CHF	4.60	10.5	3.26	<0.001
Previous stroke	9.60	13.8	8.65	0.047
Previous PCI	11.0	7.73	11.8	0.151
Previous CABG	2.15	2.76	2.01	0.568
COPD	13.6	14.9	13.3	0.646
CRF	13.8	19.9	12.4	0.012
Antiplatelet (%)				<0.001
None	4.19	8.29	3.26	
One antiplatelet*	5.52	16.6	3.01	
Combination†	90.3	75.1	93.7	
Heparin hospitalisation (%)				<0.001
None	17.5	34.8	13.5	
UFH	41.5	12.2	48.1	
LMWH	29.0	48.6	24.6	
UFH+LMWH	12.1	4.42	13.8	
ACE inhibitors (%)	57.8	47.5	60.2	0.002
Beta-blockers (%)	55.5	48.6	57.0	0.049
Diuretics (%)	39.3	51.4	36.6	<0.001
Coronariography (%)				<0.001
No	10.4	56.4	0.00	
Yes	89.1	42.5	99.6	
Major bleeding	3.16	2.90	4.42	0.405
Exitus, reinfartcion or Killip III-IV during hospitalisation	22.7	40.9	18.5	<0.001

Results with and without P-PCI.

*Only aspirin or ticlopidine or clopidogrel or others.

†Two antiplatelet drugs.

CABG, coronary artery bypass grafting; CHF, chronic heart failure; COPD, chronic obstructive pulmonary disease; CRF, chronic renal failure; GFR, glomerular filtration rate; LMWH, low molecular weight heparin; MI, myocardial Infarction; OADs, oral antidiabetic’s drugs; PCI, percutaneous coronary intervention; P-PCI, primary percutaneous coronary intervention; UFH, unfractioned heparin.

In patients who received P-PCI, the indication of medication of proven efficacy in the reduction of cardiovascular events such as beta-blockers, ACE inhibitors or double antiplatelet treatment were significantly more used. Patients with P-PCI received more heparin in acute phase but less diuretics compared with patients without P-PCI. There were no significant differences in the incidence of major bleedings between both groups. There were statistically significant differences between both groups with respect to the presentation of the combined event during hospitalisation.

Age, presence of heart failure on admission, GFR <60 mL/min at admission, previous angina, previous heart failure and previous stroke, all of them with statistically significant differences, were included in the estimation of the PS. The area under curve of the PS model was 0.68 (95% CI 0.64 to 0.73).

In the PS model, two groups of 169 patients could be included and compared. Table 2 showed the variables included in the PS in paired patients as well as the standardised differences of the variables before and after matching. There were no significant differences in these variables between the two groups. The standardised differences of the variables included in the PS decreased when matching the patients. Only the variables previous angina and glomerular filtration <60 mL/min at admission were not correctly balanced since they presented a standardised difference slightly above 10 of the corresponding SD. These two variables were included in the final model to predict the risk of the event of interest.

**Table 2:** Characteristics of patients matched by propensity score and standardised differences in propensity score variables before and after matched

Matched	n=169	n=169	P value
Age	85.0 (79.0; 88.0)	84.0 (79.0; 88.0)	0.507
Killip III-IV admission	42 (24.9%)	39 (23.1%)	0.799
GFR <60 mL/min	86 (50.9%)	77 (45.6%)	0.384
Previous angina	142 (84.0%)	148 (87.6%)	0.436
Previous CHF	153 (90.5%)	155 (91.7%)	0.848
Previous stroke	148 (87.6%)	147 (87.0%)	1.000

Standardised differences*	Before matching	After matching
Age	36.85	5.89
Killip III-IV admission	38.29	4.15
GFR <60 mL/min	24.55	10.64
Previous angina	17.71	10.15
Previous CHF	28.84	4.15
Previous stroke	16.38	1.77

*Differences between groups based on the % of the SD of each individual variable.

CHF, chronic heart failure; GFR, glomerular filtration rate.

The final logistic regression model (table 3) showed that patients who had received a P-PCI compared with those who had not received it, had a lower risk of dying or presenting with reinfarction, acute pulmonary oedema or cardiogenic shock during the hospitalisation (OR 0.55, 95% CI 0.34 to 0.89).

**Table 3:** Risk of death, reinfarction, acute pulmonary oedema or cardiogenic shock during hospitalisation in patients with STEMI aged ≥75 years undergoing P-PCI

	OR	95% CI	P value
P-PCI versus not done	0.552	0.342 to 0.892	0.015
GFR <60 mL	2.971	1.832 to 4.818	0.001
Previous angina	1.610	0.842 to 3.081	0.150

GFR, glomerular filtration rate; P-PCI, primary percutaneous coronary intervention.

## Discussion

Our study shows that the use of a P-PCI in a population of 75 years or older with a STEMI significantly reduces complications during hospitalisation, including mortality, reinfarction, pulmonary oedema and cardiogenic shock.

The elderly population that is part of ATHOS registry^8^ has an average age of 83.1 (5.9) years old with an important index of comorbidities, constituting a representative sample of the elderly patient of a European country. After an adequate characterisation and the use of a validated statistical procedure for observational studies,^12^ we have seen that P-PCI in elderly patients with STEMI reduces the risk of presenting complications during hospital admission.

Patients aged ≥75 years old with non-STEMI have received special attention in recent years,^13 14^ but more rare, and in some cases inconclusive, are the studies in patients with STEMI, particularly assessing the use of P-PCI.^15^ If we take into account the important Spanish multicentre studies published in the first decade of this century, only the TRIANA^16^ clinical trial and the ESTROFA^17^ registry included patients aged >75 years old who presented with STEMI and in whom P-PCI was performed. TRIANA showed a trend towards better survival when P-PCI was used compared with fibrinolysis, and ESTROFA described that elderly STEMI patients had a high prevalence of renal failure and multivessel disease.

The CASTUO single-centre observational study,^4^ which included patients with acute myocardial infarction, found improvement in survival in patients aged >75 when PCI was indicated during admission, both in the admission phase and during follow-up. The rest of the multicentre Spanish registries that include patients with STEMI did not study in particular the subgroup of elderly patients or the indication of P-PCI.^18 19^ Of the international multicentre studies that include both STEACS and NSTEACS, ACACIA,^20^ AMIS Plus^21^ and MINAP^22^ showed a reduction in mortality associated with invasive treatment in elderly patients with ACS both with and without ST segment elevation, although they did not analyse the P-PCI indication.

The impact and use of PCI in STEMI, between 2003and 2012 in Spain, has recently been evaluated by Cequier *et al*.^23^ In this study of 302 471 patients, 116 621 received PCI (38.6%), 46 720 fibrinolysis (15.4%) and 139 130 had no indication of reperfusion ​​(46%). The mean age of the patients who received PCI, fibrinolysis, or neither was 63.4, 63.7 and 71.8 years old, respectively. This shows a significant gradient in the age of PCI indication that penalises the elderly patients given that the mortality for the PCI group was 4.8% versus 17.3% for the group without any reperfusion therapy. The number of elderly persons is rapidly growing in Europe, and their proportion in the population is expected to increase. Elderly STEMI patients are typically treated less aggressively than younger patients because they have more comorbidities and risk of bleeding.^5–7^ For the same reasons they are also under-represented in clinical trials.

This last aspect is particularly important because it is one of the most important causes when it comes to justifying the lower indication of catheterisation and PCI in elderly patients.

This scenario constitutes a therapeutic challenge in the management of coronary artery disease elderly patients, a major concern for cardiac interventionists and surgeons. Our study shows the feasibility and benefits of intensive management with current clinical guidelines recommendations of this population, despite their high comorbidity prevalence (eg, chronic kidney failure, COPD, diabetes mellitus and previous coronary heart disease or stroke).

Among these comorbidities, the most prominent are usually the existence of diabetes, anaemia and CRF. There is a potential risk of exacerbation of CRF when the patient is submitted to a procedure that includes iodinated contrast. In addition, double antiplatelet therapy in the elderly diabetic patient and with CRF increases the risk of bleeding after PCI.^24^ These risks may explain why the indication for PCI is lower in patients aged >75 years old due to fear of complications. Savonitto *et al*^25^ analyse the importance of emphasising CRF and anaemia by carefully adjusting the dose of antithrombotic drugs and choosing a radial approach in the PCI, which results in a lower risk of subsequent bleeding.^26^ The exacerbation of CRF by contrast material and its way of preventing it continues to be a cause for concern and an unresolved discussion.^27^ Recently, the EUROTRACS study^28^ showed a reduction in mortality during hospital admission in elderly patients with ACS undergoing PCI who presented with CRF or diabetes. These results, together with the rest of the evidence, seem to indicate that, although a careful analysis of the life situation of the elderly patient should be made, the usual presence of comorbidities should not be assumed as a contraindication. An adequate clinical assessment may be sufficient to avoid depriving a patient of the benefit of a proven treatment.

The success of P-PCI has been so remarkable that recommendations have been extended to improve the application of this treatment, which is closely linked to the time of completion.^29^ In Europe, the Stent for Life Initiative^30^ has become a very active platform supporting the indications and the use of clinical guidelines in the management of myocardial infarction, defining actions that improve compliance and helping to identify barriers that may arise. In Spain, the use of reperfusion strategies has been improving year after year and, with this, mortality due to STEMI has decreased, and the incorporation of this European initiative has significantly increased the PCI rate in general and especially P-PCI.^23^

If we take into account that age is one of the variables that best predict mortality in ACS and that P-PCI is the most effective intervention to avoid it, it is clear to what extent it is important to establish its use and usefulness in the patients aged ≥75 years old who present with STEMI. We must also take into account the use of fibrinolysis as a valid alternative when times or distance prevent the realisation of a P-PCI, although there are greater bleeding risks in this population.^31 32^

Among the reasons that have traditionally been put forward to exclude those over 75 years of age and that have already been exposed, there is one,^33^ which we have not been able to explore, referring to the late arrival at the hospital of the elderly patient ‘outside the optimal window’ for perform a P-PCI. The European Society of Cardiology guidelines^5^ established that ‘a routine primary PCI strategy should be considered in patients presenting late (12–48 hours) after symptom onset’. This strategy should certainly be explored in depth.

In short, the elderly population with STEMI, due to the increase in prevalence, the benefits of P-PCI and the increased risk of bleeding secondary to the use of fibrinolytics, constitutes a group of special interest to be treated with invasive procedures.

The European Society of Cardiology Guidelines^5^ states that ‘*There is no upper age limit with respect to reperfusion, especially with primary PCI*’ but is unusual that the decision is based only in one study^16^ that could not complete the estimated sample size due to the difficulty of including patients older than 75 years. This situation, in addition to the difficulty of having sufficiently statistically powered randomised clinical trials, shows that doctors themselves have an inclusion bias, finding intangible reasons to excluding elderly patients in P-PCI prospective protocols.

Because of this, we believe that it is necessary to reinforce in daily clinical practice—that the person suffering from STEMI should always be considered to perform a P-PCI and balance the cost–benefit equation without excluding anyone by age. It is likely that in the immediate future we must refer to the elderly population as those over 85 years of age and include nonagenarians and even older.

The results of our study would support the indication of P-PCI as the treatment of choice in patients with STEMI ≥75 years old.

### Limitations

The present study has the limitations of observational studies that explore the effect of the exposure to treatments (eg, confounding, selection bias and potential reverse causation), which have been overcome by PS. The information brought up in our study is otherwise difficult to obtain in elderly patients because they tend to be excluded from clinical trials.

The relatively small sample size is another limitation. The difficulty of recruiting elderly patients is commonly referred to in the literature.

## Conclusions

The performance of P-PCI is significantly associated with a lower risk of major complications in patients ≥75 years old admitted for a STEMI.
